# Rapid Classification of Petroleum Waxes: A Vis-NIR Spectroscopy and Machine Learning Approach

**DOI:** 10.3390/foods12183362

**Published:** 2023-09-07

**Authors:** Marta Barea-Sepúlveda, José Luis P. Calle, Marta Ferreiro-González, Miguel Palma

**Affiliations:** Department of Analytical Chemistry, Faculty of Sciences, Agri-Food Campus of International Excellence (ceiA3), IVAGRO, University of Cadiz, 11510 Puerto Real, Spain; marta.barea@uca.es (M.B.-S.); joseluis.perezcalle@uca.es (J.L.P.C.); miguel.palma@uca.es (M.P.)

**Keywords:** food waxes, petroleum-derived products, paraffins, visible–near-infrared spectroscopy, machine learning, support vector machine, random forest, discrimination, *spectralprint*

## Abstract

Petroleum-derived waxes are used in the food industry as additives to provide texture and as coatings for foodstuffs such as fruits and cheeses. Therefore, food waxes are subject to strict quality controls to comply with regulations. In this research, a combination of visible and near-infrared (Vis-NIR) spectroscopy with machine learning was employed to effectively characterize two commonly marketed petroleum waxes of food interest: macrocrystalline and microcrystalline. The present study employed unsupervised machine learning algorithms like hierarchical cluster analysis (HCA) and principal component analysis (PCA) to differentiate the wax samples based on their chemical composition. Furthermore, nonparametric supervised machine learning algorithms, such as support vector machines (SVMs) and random forest (RF), were applied to the spectroscopic data for precise classification. Results from the HCA and PCA demonstrated a clear trend of grouping the wax samples according to their chemical composition. In combination with five-fold cross-validation (CV), the SVM models accurately classified all samples as either macrocrystalline or microcrystalline wax during the test phase. Similar high-performance outcomes were observed with RF models along with five-fold CV, enabling the identification of specific wavelengths that facilitate discrimination between the wax types, which also made it possible to select the wavelengths that allow discrimination of the samples to build the characteristic *spectralprint* of each type of petroleum wax. This research underscores the effectiveness of the proposed analytical method in providing fast, environmentally friendly, and cost-effective quality control for waxes. The approach offers a promising alternative to existing techniques, making it a viable option for automated quality assessment of waxes in food industrial applications.

## 1. Introduction

Petroleum waxes are a petroleum-derived product (PDP) with a wide spectrum of industrial applications obtained from lubricating oils. Within the agri-food industry, waxes are commonly used to make fruits, vegetables, and candy look shiny or as a food additive. They provide a protective layer that helps to extend shelf life by reducing the loss of water, thus slowing the dehydration process and keeping the product fresh for a longer period. This application is particularly important for fruits and vegetables that have to be transported long distances. Moreover, the wax layer can also serve as a carrier for other food additives, such as fungicides and antioxidants, further contributing to preserving the quality and safety of the produce. In chemical terms, waxes are complex mixtures composed mainly of long chains of saturated hydrocarbons (n-paraffins, isoparaffins, and cycloparaffins) and are therefore poorly reactive [1]. In addition to saturated hydrocarbons, other minor components such as aromatic hydrocarbons and sulfur- and nitrogen-containing compounds can also be included in their composition. However, due to their applications in food and cosmetic–pharmaceutical industries, waxes are subjected to a hydrotreating process [2] involving catalytic hydrogenation reactions at high pressure and temperature to remove any traces of these minor compounds to achieve the desired degree of purity, improve color, eliminate odor, and thus satisfy the requirements established by the Food and Drug Administration (FDA) [3,4] and the Pharmacopoeia of the European Union (Ph. Eur.) [5]. Petroleum-derived waxes can be classified into macrocrystalline and microcrystalline. In this sense, macrocrystalline waxes are formed from fractions of petroleum distilled under pressure and heat, and they are predominantly composed of long-chain linear hydrocarbons, mainly alkanes, with a carbon number that typically varies between 20 and 50. Due to this linear structure, macrocrystalline waxes have a harder and more brittle consistency. For its part, microcrystalline waxes are produced through the dewaxing process of lubricating oil residues. As the name suggests, these waxes have smaller crystals compared to macrocrystalline waxes since they are composed of a mixture of hydrocarbons, including branched-chain hydrocarbons and cyclic hydrocarbons. The cyclic and branched components disrupt the orderly arrangement of molecules, resulting in smaller crystals and wax with higher plasticity and flexibility [6,7]. Such differences in chemical composition allow the physicochemical properties of each type of wax and their applications to be slightly different. In this sense, macrocrystalline waxes, due to their hardness and brittleness, are mainly used in the manufacture of candles, crayons, and waterproof paper. In the agri-food industry, they find particular use as an additive in chewing gum, providing structure and contributing to its texture. Another distinct application of macrocrystalline waxes in the food industry is in the production of baked goods. They are used as release agents on baking trays and molds to ensure that the baked items do not stick, simplifying the removal process and maintaining the integrity of the products. They can also be found in the production of fruit wax coatings that are designed to enhance the visual appeal of fruits, providing a glossy appearance and serving as a barrier against moisture loss [6,8,9]. Meanwhile, microcrystalline waxes, owing to their higher plasticity and flexibility, are used to stabilize the structure of some cosmetic products (e.g., lipsticks). In the agri-food industry, they are used to coat fruits and cheeses but also uniquely contribute to the properties of various other food items. For instance, microcrystalline waxes are commonly used as a texture modifier in a variety of confectionery products, such as caramels and toffees, preventing the crystallization of sugar, improving the mouthfeel, and providing a glossy finish. Moreover, their adhesive properties are capitalized in the manufacture of cereal bars, where they help bind the ingredients together [9,10].

The quality control of waxes in the petrochemical industry is internationally regulated by the standards established by the American Society for Testing and Materials (ASTM), which are mainly based on methods used to measure physicochemical properties such as melting point (ASTM D87—Standard Test Method for Melting Point of Petroleum Wax) [11], freezing point (ASTM D938—Standard Test Method for Congealing Point of Petroleum Waxes, Including Petrolatum) [12], needle penetration (ASTM D1321—Standard Test Method for Needle Penetration of Petroleum Waxes) [13], and oil content (ASTM D721—Standard Test Method for Oil Content of Petroleum Waxes) [14]. In addition, as waxes are used for different food purposes, they must be free from undesirable odors and colors. To evaluate the odor and color, ASTM D1833—Standard Test Method for Odor of Petroleum Wax [15] and ASTM D156—Standard Test Method for Saybolt Color of Petroleum Products [16] are the protocols commonly used. However, the most accepted classification for petroleum waxes is the one defined by ASTM-TAPPI (1963) [17], which divides them based on their freezing point and their refractive index at 212 °F. In turn, gas chromatography (GC), usually coupled to mass spectrometry (MS), has also been used as a reference technique to carry out the individual identification of hydrocarbons in waxes and other PDPs [1,18,19]. Nevertheless, the target-based identification procedure of this analytical technique can be time-consuming. Therefore, considering that applications of petroleum-derived waxes vary according to their properties, having fast analytical methodologies that can be used in-line to discriminate the type of wax during the production steps becomes of great interest for the automation of the quality control process and to improve the profit margin of this product. These analytical techniques reinforce the reliability of the results as they enable the characterization of the chemical composition. Additionally, the automation involved in these methodologies implies a reduced reliance on human intervention, thus minimizing the risk of human errors. Within this framework, spectroscopic techniques such as visible and near-infrared spectroscopy (Vis-NIR), infrared spectroscopy (IR), or ultraviolet and visible spectroscopy (UV-Vis), used as screening and global profiling methods, constitute fast, environment-friendly, and on-site operation technologies that can provide an alternative to reference methods in the analysis of petroleum waxes with greater precision and repeatability.

In particular, Vis-NIR spectroscopy has proven its effectiveness in industrial and research laboratory applications over the years [20,21]. Nonetheless, the use of this spectroscopic technique generates a large amount of information in a limited period. Handling this volume of data requires the application of machine learning algorithms to transform the data into interpretable information as well as to generate predictive models that can be used to build interactive applications to automate quality control processes at the industrial level [22,23]. Thus, a suitable pretreatment of NIR spectra along with the appropriate choice of the machine learning algorithm is of great importance to achieve this objective. Several mathematical corrections (e.g., first derivative with the Savitzky–Golay method, standard normal variation (SNV), or orthogonal signal correction (OSC)) have been used to improve the quality of NIR spectra by minimizing the signal/noise and redundant contributions that can affect the performance of the machine learning models [24,25]. Regarding machine learning algorithms, numerous unsupervised and supervised algorithms are widely available. On the one hand, unsupervised machine learning techniques such as hierarchical cluster analysis (HCA) and principal component analysis (PCA) are mainly used for pattern recognition within the dataset and therefore do not allow for future predictions. Consequently, it is necessary to use supervised machine learning algorithms, such as linear discriminant analysis (LDA), support vector machine (SVM), random forest (RF), or partial least squares (PLS) to generate predictive classification and/or regression models [23,26]. In this way, Vis-NIR spectroscopy has been successfully applied in the petrochemical research sector in combination with supervised machine learning techniques, such as PLS, to quantify the removable oil content through the dewaxing process using toluene and methyl ethyl ketone (MEK) [1]. Likewise, it has been used for the quality control of lubricating oils in combination with other supervised machine learning techniques such as PLS [27]. Furthermore, this spectroscopic technique was also successfully applied for the discrimination of gasoline according to its research octane number (RON) in combination with unsupervised machine learning algorithms such as HCA, parametric supervised algorithms such as LDA and quadratic discriminant analysis (QDA), and nonparametric techniques such as SVM and RF [28,29,30].

While Vis-NIR spectroscopy and machine learning may have individual precedents, their combined application specifically for petroleum waxes, to the best of our knowledge, has not been previously developed. The synergy of Vis-NIR spectroscopy with machine learning delivers a novel analytical lens, empowering more efficient and automatic discrimination based on chemical intricacies.

Thus, based on the abovementioned review, this study aims to evaluate the applicability of a methodology based on Vis-NIR spectroscopy in combination with unsupervised machine learning algorithms (HCA and PCA) for pattern recognition and nonparametric supervised machine learning algorithms (SVM and RF) to generate predictive models for the discrimination of food petroleum-derived waxes according to their chemical composition (macrocrystalline and microcrystalline).

## 2. Materials and Methods

### 2.1. Wax Samples

A total of 60 wax samples, 36 macrocrystalline and 24 microcrystalline, supplied by Compañia Española de Petroleos, S.A.U., (CEPSA) San Roque refinery (Cadiz, Spain) and taken from different years, were used. It should be highlighted that the samples were taken from different years to increase the heterogeneity of the samples and thus enhance the reality and robustness of the analytical methods being developed. By introducing this temporal variability, the aim was to ensure that models do not overfit specific samples and remain effective when applied to a broader range of samples, potentially from different years. In Table 1, the physicochemical data provided by the manufacturer regarding the samples used in the present study are presented.

### 2.2. Sample Preparation

Before analysis, waxes (0.4 g) were stored in sealed 10 mL vials (Agilent Crosslab, Santa Clara, CA, USA) and melted in an oven at 80 °C for 10 min. Then, the samples were solidified at room temperature (25 °C) inside the same vial to obtain a plane and homogeneous solid surface that completely covers the bottom of the vial.

### 2.3. Vis-NIR Spectra Collection

The Vis-NIR spectra were recorded on a FOSS XDS Rapid Content™ analyzer with XDS near-infrared technology (FOSS Analytical, Hilleroed, Denmark) using the routine analysis software ISIscan version 6.6069.412 (FOSS Analytical). Measurements were performed using the samples stored in the sealed 10 mL vials. Empty vials were used for blank measurements. Wax samples were analyzed in the range of 400–2500 nm with a spectral resolution of 0.5 nm. A total of 32 scans per sample were collected, using the mean spectrum afterward. All samples were analyzed in duplicate. Finally, the Vis-NIR average spectrum obtained for each wax sample was placed in a Dmxn data matrix where n is the number of wax samples (*n* = 60) and m is the number of absorbance values (*m* = 4200).

### 2.4. Data Analysis

#### 2.4.1. Preprocessing

During visual inspection (Section 3.1. Spectral Analysis), Multiplicative Scatter Correction (MSC) was applied to the raw spectra for better visualization and comparison. MSC lowers the differences in the spectra produced by different light scattering and path lengths during the analyses.

In order to develop the predictive machine learning models, spectral pretreatment was carried out by obtaining the first derivative for each sample spectra using the Savitzky–Golay method with a window of 11 moving points and a third-order polynomial. The Savitzky–Golay method was chosen for its ability to preserve the heights and widths of peaks in the data, introduce minimal distortion in signals, and provide an efficient computational approach for both smoothing and differentiation. This made it an effective tool for enhancing sensitivity to spectral characteristics while eliminating noise and unwanted variations.

#### 2.4.2. Unsupervised Machine Learning Algorithms

In the present work, two unsupervised machine learning algorithms, HCA and PCA, were selected to conduct an exploratory study for finding patterns and grouping trends in the preprocessed dataset.

#### 2.4.3. Classifiers

A total of four models based on SVM and RF classification algorithms were generated and compared for the discrimination of waxes according to their chemical composition (macrocrystalline and microcrystalline waxes). To build both classifiers, the preprocessed dataset was randomly divided into a train set (split = 0.7) and a test set (split = 0.3). The train set was used during the hyperparameter optimization and training process and the test set for the validation of the generated models. On the other hand, five-fold cross-validation (CV) was used during the hyperparameter optimization and training process to minimize model overfitting. The performance of the models was carried out using accuracy and kappa as metrics. Accuracy was calculated as the percentage of correctly classified samples divided by the total number of classified samples, while kappa was computed as the difference between observed accuracy minus expected accuracy divided by 1 minus expected accuracy.

#### 2.4.4. Software

All data analysis was performed with RStudio (version 4.1.2, Boston, MA, USA). The first derivative for each sample spectrum using the Savitzky–Golay algorithm was calculated with the *savitzkyGolay* function from the *prospectr* package (version 0.2.3). HCA was performed using the *hclust* function of the *stats* package (version 4.1.2). Linkage method selection for the HCA was established by computing the agglomerative coefficient of different linkage methods (Average, Single, Complete, and Ward) using the *agnes* function of the *cluster* package (version 2.1.2). The HCA results were represented in a dendrogram using the *fviz_dend* function of the *factoextra* package (version 1.0.7). The PCA was carried out using the *prcomp* function of the *stats* package (version 4.1.2). The *fviz_eig* function of the *factoextra* package (version 1.0.7) was used to extract and visualize the result of the PCA. The scores and loadings obtained in the PCA were plotted using the *ggplot* function of the *ggplot2* package (version 3.3.5). The SVM and RF models were developed using the *caret* package (version 6.0-90). The contour plot for the SVM model was generated using the *filled.contour* function of the *graphics* package (version 4.1.2). The one-way ANOVA for the selected variables by the RF model was conducted with the *aov* function of the *stats* package (version 4.1.2). The *spectralprint* radar chart was generated using the *ggplot* function of the ggplot2 (version 3.3.5) and *ggiraphExtra* packages (version 0.3.0). The web application was developed using the *shiny* package (version 1.7.1).

## 3. Results

### 3.1. Spectral Analysis

**Figure 1:**
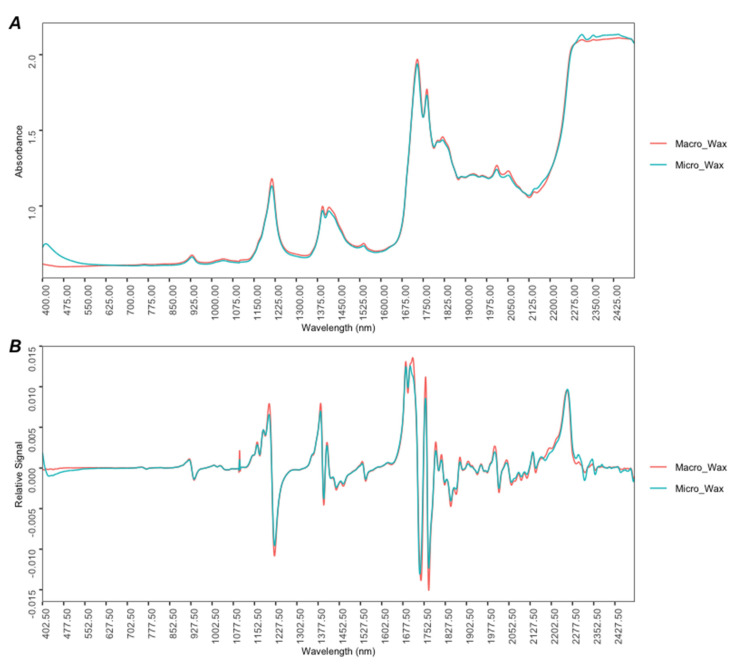
(**A**) Average Vis-NIR absorbance spectra (MSC correction; 400–2500 nm) for all wax samples studied; (**B**) Average first derivative spectra using the Savitzky–Golay method with a window of 11 moving points and a third-order polynomial for all wax samples studied.

The typical Vis-NIR spectra (MSC correction; D_4200×60_) for the two types of waxes, macrocrystalline and microcrystalline, are shown in Figure 1A. A similar profile curve was detected for both wax types. However, understanding the nuances in spectral features can offer insights into the distinct chemical structural differences. Therefore, a comprehensive visual analysis of the spectra was undertaken.

Upon thorough examination of the spectra, variations in absorbance intensity were identified in certain spectral regions. The distinct differences in absorbance intensity in the visible region, particularly at 400 nm, suggest that macrocrystalline waxes might contain specific chemical entities or configurations that differ from those in microcrystalline waxes. These entities could be tied to the violet-blue light absorbance, possibly hinting at variations in conjugated systems or distinct aromatic compounds. Furthermore, small absorption intensity differences between 800 and 1680 nm are evident in the NIR region. These NIR zones are associated with the first, second, and third overtone bands. The highest difference is found around 1200 nm and at 1375–1450 nm, which relate to the C–H 2nd overtone and the 1st overtone of C–H combinations, respectively [31]. At the spectral region around 1700 nm, the bands are characteristic of the C–H 1st overtone. Subtle differences in these bands may serve as indicators of structural variations in hydrocarbon chains, highlighting potential distinctions in saturation levels or molecular configurations between the waxes. On the other hand, the band at 2200–2400 nm, indicative of the C–H combination bands, signifies complex vibrational interactions of carbon and hydrogen, so the differences are related to different chemical compositions in the waxes [32]. This spectral behavior underscores the disparities in hydrocarbon chain lengths, levels of saturation, and branching between the two waxes—a testament to their distinct chemical structures. The spectral data acquired in this research aligns well with the study conducted by Palou et al. (2014) [1] in which NIR spectroscopy was used to analyze fully refined wax samples for evaluating MEK-removable oil. This comparison showed that in the NIR region, the spectra for the samples studied here presented a similar profile to those studied by these authors and were therefore in agreement with the literature.

Figure 1B shows the first derivative spectra using the Savitzky–Golay method with a window of 11 moving points and a third-order polynomial. The application of the Savitzky–Golay filtering method implies a slight loss of data at the beginning and end of the data matrix. Thus, it was reduced to D_4190×60_. The description of the first derivative Vis-NIR absorption spectra shows some prominent peaks in the NIR region.

Spectral analysis has uncovered some chemical differences between macrocrystalline and microcrystalline waxes. However, these differences are not easily distinguishable through mere visual inspection, making it difficult to differentiate between the two types of waxes. To enhance the accuracy of discrimination and aim for more automated interpretations, there is a need to integrate machine learning techniques with the spectral data.

### 3.2. Exploratory Study

First, an HCA was carried out to corroborate the tendency of wax samples to group according to their chemical composition. HCA is one of the most popular clustering methods in data science and constitutes an unsupervised machine learning technique that allows grouping a set of data into subsets or clusters based on their similarity, merging the two closest clusters into a larger one [33,34]. This unsupervised machine learning technique was applied to the preprocessed dataset (D_4190×60_).

**Figure 2:**
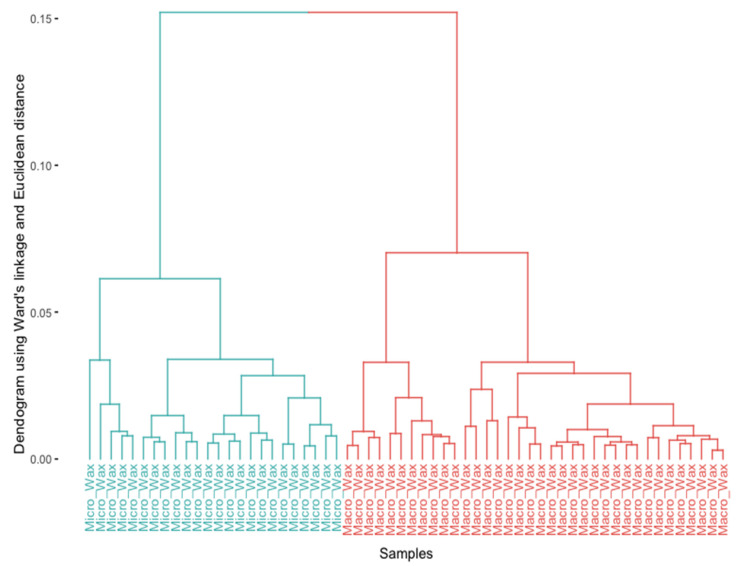
Dendrogram obtained from the HCA using the Euclidean distance and Ward’s method. The wax samples (*n* = 60) are colored according to their chemical composition: light blue for microcrystalline waxes and light red for macrocrystalline waxes.

For this analysis, Euclidean distance was selected as the distance measure and Ward’s method as the linkage method. The choice of the linkage method was determined based on a comparison of the agglomerative coefficient of different methods (Average, Complete, Single, and Ward). Agglomerative coefficients close to 1 would indicate a stronger clustering structure. In this case, the Ward method presented the highest agglomerative coefficient (0.95) among the linkage methods evaluated. The results obtained through HCA are plotted in the dendrogram shown in Figure 2. According to the results, it can be observed that the samples tend to group into two main clusters: on the one hand, the cluster with a light blue color is completely formed by all of the microcrystalline wax samples; on the other hand, all of the macrocrystalline wax samples constitute the cluster with light red color. Thus, the results indicate that there is a strong tendency to group the waxes according to their chemical composition.

**Figure 3:**
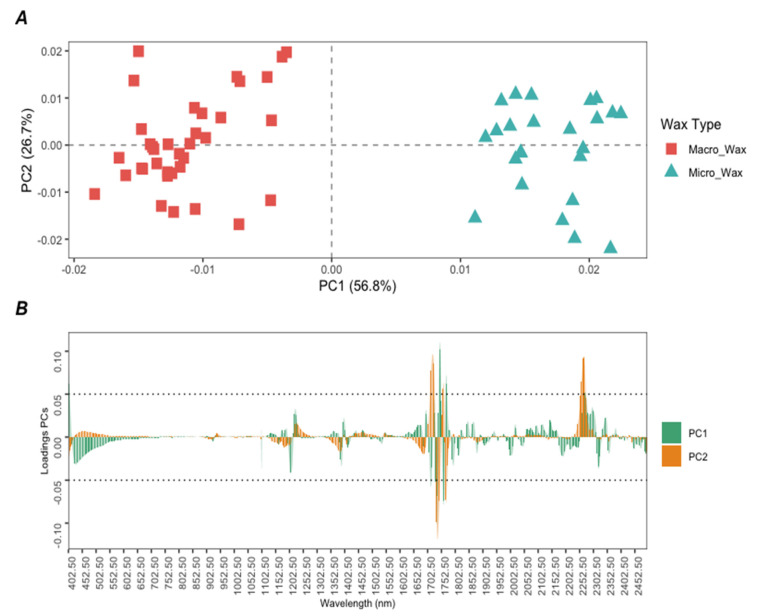
(**A**) Score plot PC1 vs. PC2 for all wax samples (*n* = 60); (**B**) Loadings obtained for each wavelength in the first two PCs in the PCA.

A PCA was performed to obtain additional information about the spectroscopic ranges producing the previous results. PCA is another unsupervised technique used in machine learning as part of the exploratory analysis of the data. This technique reduces dimensionality by losing the least amount of information possible. In this sense, it represents the variance of the data using a smaller number of uncorrelated variables, referred to as principal components (PCs), which are calculated as linear combinations of the original variables [35,36]. This unsupervised machine learning technique was also applied to the preprocessed dataset (D_4190x60_). Figure 3A shows a plot of the scores obtained by all samples (*n* = 60) for the first two PCs (PC1 and PC2), and Figure 3B represents the loadings obtained for PC1 and PC2. Both PC1 and PC2 explained 56.8% and 26.7% of the variance of the data, respectively, resulting in a cumulative variance of 83.5%. In this case, both PCs were responsible for the separation of the wax samples according to their chemical composition, obtaining two main groups. It can be observed (Figure 3A) that the microcrystalline wax samples (light blue) are separated from the macrocrystalline samples (red light), with the former having positive scores for PC1 and the latter having negative scores for this PC. The observed grouping trend was in concordance with that obtained through HCA. On the other hand, according to the loadings plot in Figure 3B for PC1 (light green), it is possible to obtain information about the spectroscopic ranges responsible for this separation. Based on the results, certain spectral regions that seem to be important can be appreciated, highlighting the highest loadings in PC1 at several wavelengths in the range of 1700 nm, associated with the C–H 1st overtone, which, as seen by visual inspection of the first derivative spectra in Figure 1A, suggests the presence of certain hydrocarbon configurations but also underscores disparities in hydrocarbon chain lengths, saturation levels, and branching between wax types.

The HCA and PCA analyses were both successful in achieving a complete distribution of petroleum wax samples according to their chemical composition. Nevertheless, these unsupervised techniques do not allow for predicting future observations. From an industrial point of view, being able to rely on predictive models is of great interest for data handling process automation. Therefore, SVM and RF algorithms were selected as nonparametric techniques to generate predictive models for the discrimination of macrocrystalline and microcrystalline waxes.

### 3.3. Support Vector Machine Classifiers

SVM is a nonparametric supervised machine learning algorithm that can handle both linearly and nonlinearly separable data. Over the years, it has proven to be one of the best classifiers for a wide range of situations, becoming a benchmark in this artificial intelligence discipline. However, in support vector regression (SVR), its application also extends to regression problems. When it comes to classification, the main idea of the linear SVM algorithm is to find the optimal hyperplane (decision boundary) so that the distance (margin) between it and the support vectors (data points of each class closest to the hyperplane) is maximum. In contrast, when nonlinear boundaries between classes are encountered, SVM can be extended to nonlinear classification by using kernel functions. The kernel function can be defined as a function that quantifies the similarity between two classes in a new dimensional space. Among all of the existing ones, the Gaussian kernel is the most used since almost any boundary shape can be obtained, and, in general, good performance is achieved [37,38,39]. In this study, SVM was used with the linear and Gaussian kernel implementation. Therefore, the optimization of the hyperparameters C (regularization parameter in linear and Gaussian kernel SVM) and σ (Gaussian kernel parameter) is an important step to consider during the construction of the SVM models since the former controls the balance of the error penalty and the latter controls the curvature of the decision boundary (Equation (1)) [40]. To carry out this optimization, there are different methods, such as the grid search or the gradient descent algorithm, usually based on the CV classification rate, to evaluate the performance of the model and minimize the risk of overfitting [41].

*Gaussian kernel*: *K*(*x*, *u*) = *exp*(−σ ||*x* − *u*||^2^) (1)

First, for the construction of the SVM classifier with the Gaussian kernel implementation, the optimization of the hyperparameters (C and σ) was performed using the train set. Thus, the grid search method with the exponential growth of C and σ was selected. In this case, log_2_C and log_2_σ were in the range of –10 to 10 in intervals of 0.5. Each combination of parameter choices was tested using a five-fold CV, and the smallest parameters that presented the best five-fold CV accuracy were selected. The smallest parameters are selected since when using the caret package in R to optimize hyperparameters, the objective is to identify parameter values that result in the best average performance on test datasets using a five-fold CV. In cases where different hyperparameter combinations produce equal performance, caret defaults to selecting the simplest model with lower hyperparameter values. Simpler models are preferred because they are less prone to overfitting, which occurs when a model is too complex and performs well on training data but poorly on new data. By selecting the simplest model with the best cross-validation score, we aim to achieve better generalization on new data. Furthermore, simpler models require fewer computational resources for training and prediction, offering advantages such as reduced computation time, lower memory usage, and minimized power consumption. This is especially beneficial in resource-constrained environments.

**Figure 4:**
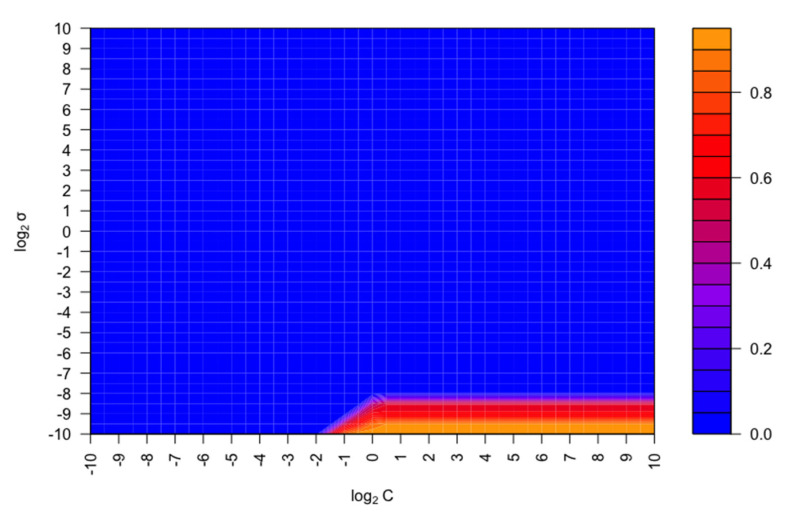
Contour plot for the five-fold CV accuracy results for the best C and σ search.

Figure 4 shows the contour plot for the search of the values of C and σ that provided the best five-fold CV accuracy. It can be observed that as the log_2_C, and hence C, increased, the five-fold CV accuracy was higher (Figure 4). A larger value of C means a smaller width of the margin and, therefore, fewer observations will violate it. As this hyperparameter controls the balance between bias and variance, a higher value of C will imply a classifier with a lower bias but a higher variance. For this reason, the optimal value of C was set to 0.7071 (log_2_C = −0.5), as it was the minimum value that allowed for a maximum of five-fold CV accuracy. In this way, an excellent performance (lower bias) was obtained, and overfitting was avoided (lower variance), corroborating the robustness of the generated Gaussian kernel SVM classifier. Moreover, the number of SVs was set to 40. On the other hand, as can be seen in Figure 4, the five-fold CV accuracy increases as the value of log_2_σ, and consequently of σ, decreases. The value of σ controls the behavior of the kernel and, as its value increases, so does the flexibility of the model. In this case, the optimal σ value was set to 9.766 × 10^−4^ (log_2_σ = −10). It should be highlighted that the best results were obtained with the lowest σ values, suggesting a more linear boundary [42]. After the hyperparameter tuning, a model was trained with the optimal values of C and σ obtained using the training set and applying a five-fold CV, obtaining a five-fold CV accuracy of 97.8% and a five-fold CV kappa of 0.95. Finally, the performance of the generated Gaussian kernel SVM classifier was evaluated using the test set. The test set showed 100% accuracy and a kappa of 1, confirming the excellent performance of the model for discriminating waxes according to their chemical composition.

The Gaussian kernel SVM model obtained reliable performance and precision for the classification of petroleum waxes. However, since the σ value suggested a linear separation of the classes, the implementation of a linear SVM model was explored to evaluate its performance in the discrimination of macrocrystalline and microcrystalline waxes, as the training time of a linear SVM model is smaller compared to the Gaussian kernel SVM one, a fact that is interesting from a model implementation point of view. In this sense, when a linear SVM is applied, the only hyperparameter to be optimized is the value of C. Therefore, the optimization of this hyperparameter was carried out using the grid search method with the exponential growth of C. Here, log_2_C ranged from −10 to 10 in intervals of 0.5. As in the previous case, each combination of C parameter options was checked using a five-fold CV, and the one with the best accuracy was selected. The lowest value of C allowing us to achieve the maximum five-fold CV accuracy value was 9.766 × 10^−4^ (log_2_C = −10). Moreover, the number of SVs was set to 9. The optimized hyperparameter was then used for training the linear SVM model, obtaining 100% five-fold CV accuracy and a five-fold CV kappa value equal to 1. The evaluation of the developed linear SVM model was carried out using the test set, resulting in 100% accuracy and a kappa value of 1. Thus, the linear SVM model developed demonstrated its robustness and good performance. These results indicate a slight improvement in the model’s performance during training using the SVM algorithm with linear implementation, so it would confirm the separability of the classes linearly. However, similar results were obtained for the performance of the model using the test set. Accordingly, the SVM algorithm with both implementations proved to be suitable for petroleum wax discrimination.

### 3.4. Random Forest Classifiers

**Figure 5:**
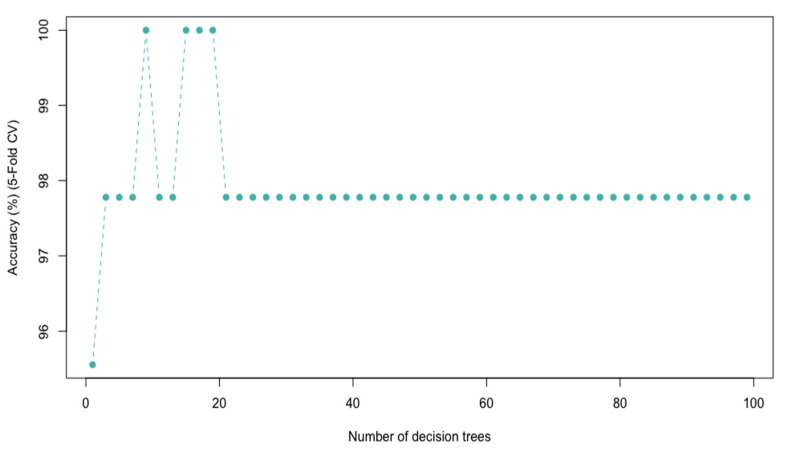
The RF performance and 5-fold CV accuracy according to the number of decision trees.

RF is a bagging-type ensemble algorithm included in the supervised machine learning algorithms that have been widely used in classification and regression problems in various sectors. The algorithm works by concurrently training several decision trees on several random subsets generated by bootstrapping followed by an aggregation (jointly called bagging). Bootstrapping means that, randomly, 67% of the original data is used to train the model (*in-bag* set), and the remaining 33% (*out-of-bag* set) is used for internal CV, evaluating the performance of the RF model through the *out-of-bag* (OOB) *error*. In practice, the RF classifier has few hyperparameters to optimize. In this case, the hyperparameter *mtry* (number of predictors randomly selected before the cutting of each tree) must be optimized. For classification, the square root of the total number of predictors is generally used as the optimal value of *mtry*. Furthermore, a specific number of decision trees in the RF model needs to be set [40]. First, the optimal hyperparameter values were established to build the RF model. The square root of the total number of predictors was used as the optimum value of *mtry* and equaled 64.73 (4200 predictors). On the other hand, in RF, the number of decision trees is not a critical hyperparameter since adding more decision trees does not imply overfitting risks and improves the performance of the model. However, its value must be determined in advance to stabilize the error and minimize the computational resource loss. To determine the number of decision trees to use, the values of *ntree* in this study were set from 2 to 100 with an interval of 2 trees, and five-fold CV accuracy was considered as an evaluation criterion. The results are shown graphically in Figure 5. As can be seen, the accuracy rate tends to stabilize at 22 decision trees and is maintained up to 100 decision trees. In this sense, the number of decision trees was set at 100 as it is a high enough number to stabilize the error without involving significant computational costs. The optimal values established for *mtry* and the number of decision trees were then used to train the RF model with the training set applying a five-fold CV. Great results were obtained for the five-fold CV with an accuracy of 97.8% and a kappa of 0.95. In addition, the OOB estimate of error rate equaled 2.33%. Subsequently, the performance of the model was evaluated using the test set, obtaining 100% accuracy and a kappa of 1. This confirmed that a reliable and accurate RF model was obtained for the discrimination of petroleum waxes in terms of their chemical composition.

Besides having reliable predictive models, one of the goals pursued when applying global profiling techniques for quality control process automation is finding a reduced set of signals that characterize the samples and allow them to be easily differentiated. The SVM, due to the nature of the algorithm itself, does not allow for establishing the most relevant wavelengths directly related to the discrimination of macrocrystalline and microcrystalline waxes. However, the RF enables this task to be performed. Here, the *varImp* function of the caret package was used to estimate each variable’s contribution to the model. In the case of RF, this function calculates the prediction accuracy in each tree’s out-of-bag portion of the data. Subsequently, the same is performed after permuting each predictor variable. The difference between the two accuracies is finally averaged across all trees and normalized by the standard error [43]. Specifically, seven wavelengths (1174.5 nm, 1751.0 nm, 1810.5 nm, 1928.5 nm, 2041.0 nm, 2117.00 nm, and 2189.5 nm) with a relative importance of more than 70% (Supplementary Figure S1) were selected for the construction of the *spectralprint*. Among the variables selected by the RF model, the wavelength of 1751.00 nm was found, which, in the PCA, presented a greater weight in the loadings. To delve deeper into the relationship between spectral and structural differences, we examined these wavelengths in more detail. The band found around 1200 nm could be linked to C-H stretching in longer-chain hydrocarbons found in the waxes, hinting at the differences in chain lengths between the two waxes. The band around 1600–18,000 nm is related to variations in branching in the hydrocarbon chains, therefore showing different kinds of hydrocarbon chains. The differences in the signals in the 2200–2400 nm range could be related also to differences in the hydrocarbon chains, as they are related to the vibrational interaction of the C-H chemical bonds.

A one-way ANOVA was performed for each of the variables selected by the RF model, and all of them were statistically significant at a 95% confidence level. Aiming to assess the feasibility of developing a *spectralprint*, the stability of the RF algorithm was studied when using the training and test sets reduced to these seven wavelengths. For this purpose, a new RF model was developed using only these selected variables. Here, the *mtry* value was set to 2.646 and the number of decision trees to 100. The result showed 100% accuracy and a kappa of 1 for the five-fold CV set, with an OOB error rate of 0%. Furthermore, the performance of the model was evaluated using the variable-reduced test set, obtaining 100% accuracy and a kappa of 1. The slight improvement in five-fold CV accuracy and kappa and OOB error rate should be highlighted. This improvement could be explained by the reduction in redundant information and noise in the dataset when performing the extraction of the selected variables. Nevertheless, since the difference between the performance is relatively minimal, both models—using 4200 and 7 wavelengths—were considered stable. Therefore, the seven selected wavelengths were used for the construction of the characteristic *spectralprint* of both types of waxes.

**Figure 6:**
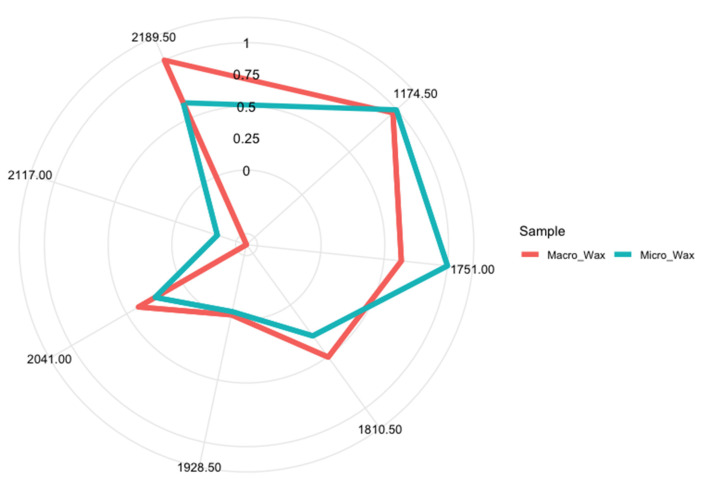
Graphical display of the characteristic *spectralprint* for each type of petroleum wax.

The radar charts for the *spectralprints* of macrocrystalline and microcrystalline waxes are shown in Figure 6. The mean values of the seven variables in each group were normalized to the maximum first derivative values. According to the results obtained, a similar *spectralprint* shape can be observed. However, differences in terms of absorbance can be detected for both types of petroleum waxes. On the one hand, macrocrystalline waxes showed their maximum absorbance at a wavelength of 2189.5 nm, whereas the absorbance percentage for this wavelength in microcrystalline wax is 0.7 (70% of the absorbance maximum). In turn, it was possible to appreciate that there were other wavelengths for macrocrystalline wax equal to or above 50% of the maximum absorbance, specifically at 1174.5 nm, 1751.0 nm, and 1810.5 nm. The remaining wavelengths were below 0.5 (50% of the maximum absorbance). On the other hand, for microcrystalline waxes, the maximum absorbance was reached at the wavelength of 1751.0 nm, while the absorbance percentage for this wavelength in macrocrystalline waxes is 0.7. In addition, other wavelengths whose intensities were found to be above 50% of the maximum absorbance could be observed for microcrystalline wax, with these being at 1174.5 nm and 2189.5 nm. The remaining wavelengths had a lower contribution (<50% of the maximum absorbance) in terms of intensity in the *spectralprint* of the microcrystalline waxes, highlighting the wavelengths at 1810.5 nm, 1928.5 nm, and 2041.00, which were above 0.5 for macrocrystalline waxes. As the intensities and the ratios of the signals are different for each type of wax, thus giving different fingerprints, these can be used for the discrimination of the different waxes based on their chemical composition in a fast and easy way.

Considering the excellent results obtained for the models developed and to make them available to users, a web application has been designed in Shiny with the RF model. The availability of web applications that support these models facilitates the processing of the data and saves time and effort in its interpretation. Access to this demo application is available through the link available in the Supplementary Materials.

### 3.5. Repeatability and Intermediate Precision

The analytical properties of the Vis-NIR-pretreated spectra, namely repeatability and intermediate precision, were established. A total of 18 analyses were performed using a macrocrystalline wax sample. Repeatability was studied by performing nine analyses on the same day, and intermediate precision was evaluated by performing three analyses per day over three consecutive days. The intra-day and inter-day coefficient of variation (C.V.) of the analysis was calculated. For this purpose, the mean of the C.V. obtained in the whole set of spectra was calculated. The obtained C.V. for repeatability was 2.43% and for intermediate precision was 2.89%.

## 4. Conclusions

Vis-NIR combined with machine learning tools proved to be a suitable practical methodology for the characterization and discrimination of petroleum waxes of agri-food interest based on their chemical composition, offering a fast, reliable, and environmentally friendly alternative to the established official methods. The results obtained through the application of unsupervised machine learning algorithms, such as HCA and PCA, suggested a strong tendency to distribute samples according to whether they were macrocrystalline or microcrystalline waxes. Supervised machine learning models were applied using the SVM with the linear and Gaussian kernel implementation and the RF algorithms. The models developed based on both algorithms have proven their effectiveness and robustness for the discrimination of the two common commercialized petroleum waxes, obtaining an excellent performance (100% accuracy on the test set). In turn, the RF model allowed the extraction of the seven most relevant wavelengths in this discrimination. Therefore, the characteristic *spectralprint* for each type was constructed. Accordingly, these *spectralprints* can be used as a suitable routine method for the rapid, accurate, and straightforward identification of petroleum wax types. Furthermore, website applications with support for computers and tablets from the generated models could be built (i.e., with Shiny), thus simplifying and automatizing the data analysis within the production chain. This, along with the portability of the analytical technique and its user-friendliness, would enable even simpler and more automated quality control of this PDP.

## Figures and Tables

**Table 1:** Physicochemical data provided by the manufacturer for macrocrystalline and microcrystalline waxes.

	Saybolt Color	Oil Content (%)	Freezing Point (°C)	Needle Penetration
Microcrystalline waxes	+10 (Minimum)	1.5 (Maximum)	71–75	25 (Maximum)
Macrocrystalline Waxes	+25 (Minimum)	1.0 (Maximum)	66–69	20 (Maximum)

## Data Availability

The data used to support the findings of this study can be made available by the corresponding author upon request.

## Supplementary Materials

The following supporting information can be downloaded at: https://www.mdpi.com/article/10.3390/foods12183362/s1, Figure S1: Graphical display of the 20 most important RF model features and their relative importance. Link to access the demo application for the automatic characterization of waxes: https://marta-barea.shinyapps.io/waxes_types_app/. To begin working with the application, the Vis-NIR spectra data file must be uploaded. Both .csv/txt and .xlsx/xls formats are accepted by the program. On the “*Download*” button, a test dataset has been attached to perform a trial run. Once the file is uploaded in the “*Browse*” tab, a window will appear just below indicating the status. After the loading is complete, it is possible to make predictions on the dataset by clicking on the “*Submit*” button. The software will directly perform the first derivative and the Savitzky–Golay filter to predict the wax type by using the RF model.
