# Supplementary material for: Rapid Classification of Petroleum Waxes: A Vis-NIR Spectroscopy and Machine Learning Approach
Source: Foods. 2023 Sep 7;12(18):3362. doi: 10.3390/foods12183362 (PMC10528079; doi:10.3390/foods12183362)
Supplement: Supplementary file 1 [file foods-12-03362-s001.zip › foods-12-03362-s001.pdf]

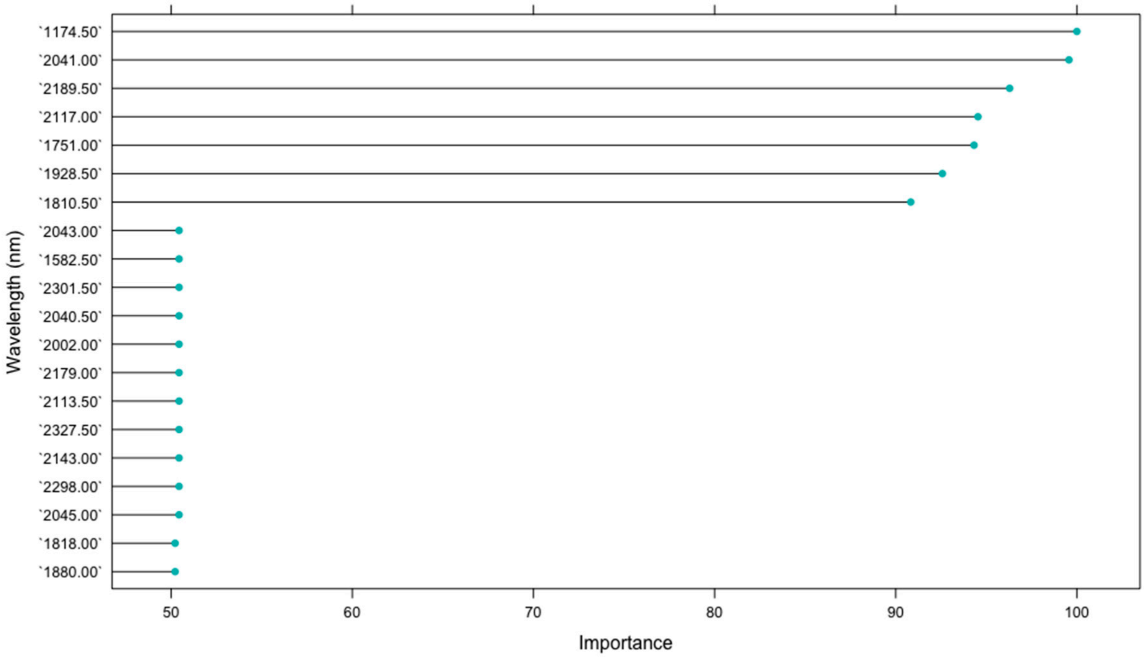

Figure S1: Graphical display of the 20 most important RF model features and their relative importance.
